# Environmental Historical Geographies

**DOI:** 10.1111/gec3.70013

**Published:** 2025-01-21

**Authors:** Matthew J. Hannaford

**Affiliations:** ^1^ Department of Geography University of Lincoln Lincoln UK

**Keywords:** climate history, environmental history, environmental impacts, environmental interventions, environmental knowledges, environmental reconstruction, historical geography

## Abstract

Environmental historical geography is a diverse, dynamic and active subfield with close connections to environmental history. Here, I examine developments in three overarching and overlapping themes within the subfield: environmental reconstruction, environmental knowledges and discourses, and environmental impacts and interventions. For each area, I highlight recent approaches to, and applications of, environmental historical geography. I also draw attention to several promising areas of research where environmental historical geography can build on its existing strengths and continue reinvigorating understanding of environment‐society relations. These include contextualising environmental knowledge and data production amidst advances in big data and AI; illuminating the multi‐directional interactions between environmental change, knowledges, and materialities; revealing the entangled physical and intellectual legacies of imperial and colonial projects; and enhancing comparative research.

## 1 Introduction

The urgent and complex challenges engendered by human impacts on the environment and environmental impacts on humans have prompted ever‐growing scholarly interest in past human‐environment interactions. This has emerged from various disciplinary centres, hence research under this broad umbrella has addressed a multiplicity of aims and adopted diverse approaches resting on varying epistemological foundations. One such area is environmental historical geography—a diverse, dynamic and active subfield stemming from the subdiscipline of historical geography, but with considerable overlap with cognate subfields of environmental history, historical ecology, historical climatology, and historical political ecology. Key aspects span reconstructions of past environments—an endeavour with roots in physical geography –, examination of environmental knowledges and ideas, which emerged more firmly from the historical‐cultural geography tradition, and investigation of material impacts and interventions. The contribution of this multifaceted research in environmental historical geography to analyses of human‐environment interactions forms the focus of this article.

There exist various wide‐ranging attempts to take stock of research in environmental historical geography, published at intervals over the past three decades (Williams 1994; Baker 2003; Colten et al. 2003; Wynn et al. 2014). Each of these surveys have identified a set of key themes and topics in the field (Table 1). These range from the three overarching themes identified by Baker (2003) to the six more topic‐focused areas pinpointed by Craig Colton (Wynn et al. 2014). My focus on themes rather than topics is closer to Baker's analysis, however I broaden the latter two of his categories (impacts of humans on natural environments, and human perceptions of past environment) to account for work conducted in recent years. Specifically, I engage with developments and debates in (i) environmental reconstruction, (ii) environmental knowledges and discourses, and (iii) environmental impacts and interventions. In doing so, I highlight recent approaches to, and applications of, environmental historical geography, as well as draw attention to some areas for future research. Although each area is considered distinctively, I draw connections across themes, and a closing argument of the paper is that the multi‐directional interactions between environmental changes, environmental knowledges and human interventions should be an area for future work.

**TABLE 1:** Key themes and topics in environmental historical geography.

Author (year)	Themes and topics	Examples
Williams (1994)	Human transformation and modification of the earth	Cultures as agents of modification in climate, soils, plants, animals, waters, landforms
	Global expansion and the capitalist economy	Imperialism and colonialism, industrial capitalism, resource exploitation
	The place of humans in nature	Impact of environmental ideas on thought and action (e.g., conservation and environmental action)
	Interrelationships among habitat, economy, and society	Perception, adjustment and management response to natural hazards, agricultural systems, demography, ecological sustainability
Baker (2003)	Reconstructing past physical environments	Climate, glaciers, vegetation, fauna
	Impacts of humans on natural environments	Vegetation (e.g., fires, grazing, deforestation), animals (e.g., invasive species), soils (e.g., salinisation, erosion), water (e.g., water management, river modification, wetland reclamation), landforms, climate and atmosphere
	Human perceptions of past environments	Past geographical and environmental knowledge, constructions of wilderness
Colten et al. (2003)	Relationship between people and material environmental changes	Ecosystem change, geomorphology (e.g., impact of human activities on weathering and erosion), biogeography, transformation of rural environments, urban geographies
	Attitudes, values and ideas associated with material environmental change	Rural and wild areas, urban environments
	Changing politics of the environment	Social conflicts, policy development, mismanaged environments, development of natural resource agencies
Colten in Wynn et al. (2014)	Parks and protected areas	Management of situated resources, delineation of wildlife refuges, notions of place, urban parks and land use
	Resource valuation and use	Policy shifts and pollution, wetland drainage, water resources conflict, traditional water management, re‐evaluation of resources in colonial societies
	Urban issues	Hazardous waste disposal, NIMBYism, race and urban land use, urban environmental justice
	Climate change	Reconstruction of past climates, human vulnerability and adaptation, policy initiatives
	Hazards	Social vulnerabilities to hazards, resilience, memory
	Historical cultural ecology	Human impacts and alteration of the physical landscape

Since the earlier classifications in Table 1 were published, current issues such as climate have risen swiftly up the agenda in environmental historical geographical enquiry, as illustrated by Offen's (2014) third *Progress in Human Geography* report dedicated to the topic and a deluge of further scholarship since then. Climate forms a crucial part of this article, alongside other environmental classifications and issues. A further area of attention (and often contention) that I engage with is the ‘call for relevancy’ emerging from institutional environments that increasingly value and incentivise the practical and policy implications of research (Offen 2012). Rather than viewing this as a threat that historical geographers are forced to confront, I argue that the discipline has and continues to provide myriad critical insights into environment‐society relations that are of benefit to society.

Before beginning, it should be restated that the boundaries of environmental historical geography are loose. Several interventions have pointed towards the inherent synergies between (environmental) historical geography, environmental history and historical ecology (Colten 1998; McNeill 2003; Naylor 2006; Wynn et al. 2014). While I certainly seek to highlight the contribution of geographers within the shared endeavours of tracing past environment‐society interactions, I do not exclude geographically oriented research emerging from other disciplines. Rather, I focus on themes and debates that span these disciplines and identify points of intersection instead of dwelling on points of distinctiveness or pursuing calls for more integration. As argued by the environmental historian Martin Melosi, ‘to speak about a dichotomy between geography and history (or any two disciplines) is to constrain intellectual horizons. Equally, to argue for the necessity of connection is useful but limiting’ (Wynn et al. 2014, 14). Two final delimitations are necessary. First, while the scope of this article is global, the focus is largely on English‐language literature. Second, emphasis is mostly (but not exclusively) placed on literature engaging with written evidence, although related sources (field evidence) and data (climate reanalyses) are considered in conjunction.

## 2 Environmental Reconstruction

Efforts to reconstruct and characterise past environmental conditions and changes using the ‘archives of society’ have been a key feature of work by geographers since the 1970s. However, with the exception of Baker (2003), these have not featured heavily in previous stock‐takes of environmental historical geography, but have more commonly been considered alongside reconstruction efforts from the ‘archives of nature’ central to the palaeosciences (PAGES2k Consortium 2017). Climate and weather have received most attention within this endeavour, partly owing to the urgency of understanding climate variability and change over long timescales, but also due to their prevalence in diverse historical source types long before instrumental meteorological records began. Other hazards that have received significant attention include cyclones and seismic hazards. Further reconstructions relate to biodiversity and land use, although this work has tended to be more dispersed.

Climate reconstruction from historical sources has taken place at local to global scales. Much research has been conducted at the local to regional level and has made use of bodies of written records to classify climatic conditions (temperature, precipitation, floods, drought, snow/ice, and wind) at monthly, seasonal and/or annual timescales. In Europe, Latin America and the Caribbean, southern Africa, China, India, and Australia, reconstructions of temperature and precipitation have analysed narrative evidence using content and textual analysis to produce ordinal climate indices (Nash et al. 2021). In addition, geographers have been at the forefront of developing reconstructions from records of lake freezing dates and phenological records such as cherry blossom, particularly in Japan where such records are of great length (Mikami 2023). Geographers have also led reflective methodological work that examines divergence of practice (Nash et al. 2021) and the influence of researcher subjectivity on the reliability of reconstructions from narrative material (Adamson, Nash, and Grab 2022), finding them to be highly reliable. While the global distribution of reconstructions has undergone a significant increase in recent years (Burgdorf et al. 2023), source collections in numerous parts of the world remain underutilised—for example, western Africa, southeast Asia, the Middle East and the Pacific.

Reconstructions of (daily) weather can help enhance understanding of the mechanisms and impacts of extreme weather and decadal to multi‐decadal variability, but have yet to receive significant attention from geographers. Prior to the creation of national meteorological networks, such reconstructions are reliant on sources that are innately local and available at daily resolution, particularly diaries (Adamson 2015). Daily weather reconstructions have received less attention than seasonal or annual scale climate reconstructions (although see Manley 1975; Chenoweth et al. 2007; Wheeler et al. 2009). However, increased interest in extreme weather by climate scientists and advances in historical climate reanalyses (Slivinski et al. 2019) have created renewed drivers for this work. Environmental historical geographers could usefully contribute to these efforts by revisiting archives, digitising (sub)‐daily data and building assemblages of daily data that can enhance and extend weather reconstructions. Brönnimann, for example, suggests that daily weather reconstructions may be possible back to the late‐seventeenth century in Europe (Brönnimann 2023), while elsewhere valuable advances could be made by conducting similar work with daily records kept during the late‐19th and early‐20th centuries.

A major contributor to the growing amount of daily data available for reanalyses have been citizen science ‘data rescue’ projects (e.g., Brönnimann et al. 2018; Hawkins et al. 2019, 2023). Such projects have accurately transcribed millions of observations for scientific analysis and integration into weather and climate models and services. As data rescue projects continue apace and increasingly become supplemented by AI, it is important for historical geographers to collaborate with climate scientists to unpack the historical geographies underpinning the data, but also to enhance data accessibility for scholars outside of the sciences. Publicly accessible databases developed by teams of geographers and historians such as Tambora (Riemann et al. 2015) and TEMPEST (Veale et al. 2017) provide examples that largely comprise qualitative entries. Yet many numerical series of observations also contain narrative remarks that often possess valuable insights into the relationships between shifting environmental knowledges and extreme events, and it is crucial that these narratives are not lost amidst the drive for measurable, comparable data. This is an important part of what Sieber et al. (2022) identify as formalising trust in historical weather data. In this sense, Brönnimann et al. (2018) sensibly call for a ‘distributed approach’ to data rescue—in other words, one that is shared amongst diverse research projects and builds engagement between scientists and researchers in the humanities and social sciences. This could help bridge the gap between the different forms of knowledge production inherent in environmental reconstruction and research discussed in the following section.

There is a long history of reconstructions of environmental hazards beyond climate and weather, which have tended to take the form of catalogues and classifications of extreme events. The history of hurricanes has received significant attention in the Caribbean and North Atlantic, where occurrence has been catalogued and analysed back to 1494 CE (Chenoweth and Howard 2023). This work has extended to other regions of the world, including the Indian Ocean (Nash et al. 2015; Msemo, Finney, and Mbuya 2022), though more remains to be done in extending geographical coverage and comprehensiveness. Earthquake and tsunami reconstructions arguably represent one of the most multidisciplinary areas of work in this sphere. Much of this has been led by geologists, geophysicists and seismologists, however the integration of historical and natural archives is a distinguishing feature of this research (Kaabouben et al. 2009; Papadopoulos et al. 2011; Maramai, Brizuela, and Graziani 2014; Stucchi et al. 2013). Disciplinary crossovers have also been central to the reconstruction of historical changes in wildlife distribution and abundance—in this case between historical geography and historical ecology (Allen and Keay 2006; Santrůčková, Dostálek, and Demková 2015; Grab and Nash 2022).

Another aspect of historical environmental reconstruction pertains to land use and land cover, which represent anthropogenic forcings integral to climate models. Developing reconstructions of past land cover and land use change was the goal of the Past Global Changes (PAGES) LandCover6k project (Morrison et al. 2021), yet while archaeology and the palaeosciences were at the forefront of this work, the participation of historical geographers and environmental historians was more limited (although see related research, e.g., Widgren 2018; Sluyter 2021; Hannaford 2023). As Mauelshagen (2014, 18) argues, ‘archival documents related to land use, desertification, de‐ and reforestation are pieces in the same puzzle of anthropogenic environmental changes that may affect local or global climate changes’, however so far relatively little has been done to exploit the potential of these sources to develop systematic reconstructions of land use and land cover. The importance of examining intersections between environmental knowledges and the observations that underpin environmental reconstructions is expanded on in the next section. Reconstruction of past environments also provides crucial context for work on human impacts and interventions, which is discussed in Section 4.

## 3 Environmental Knowledges and Discourses

Alongside other subdisciplines, historical geography has a long tradition of examining the emergence and evolution of environmental knowledges within different geographical and temporal settings (Glacken 1967; Livingstone 1995, 2024; Endfield and Morris 2012; Carey 2012). This trend has ‘animated research in historical geography like few other developments in recent memory’ (Offen 2012, 530) and stands in contrast to the more dispersed multidisciplinary historical geographical work on environmental reconstruction. This work remains relatively separate from that discussed in the previous section, largely due to tensions between divergent epistemological underpinnings. A related endeavour is the development of genealogies of scientific knowledge and concepts, especially those pertaining to climate. A further rapidly growing focus is the role of imperialism and colonialism as forces that remade environmental understandings and meanings. On the flip side, a topic that has come to the fore more recently is the role of indigenous and local knowledges in shaping the construction and production of scientific discourse in the age of empire. Such work is important not only as context but also in revealing how responses to environmental change today are constitutive of past geographies and uneven power relations.

The need to ‘particularise’ (and hence pluralise) environmental knowledges, meanings and experiences within place‐time contexts has been a central concern (Endfield and Morris 2012; Golinski 2007; Jankovic 2010). This has often been situated as a counterpoint to the abstractness of scientific discourse on climate change, instead centring everyday experience, memory and sense of place in lay discourses and understandings of weather and climate (Brace and Geoghegan 2011; Geoghegan and Leyson 2012; Veale, Endfield, and Naylor 2014; Hall and Endfield 2016). Endfield (2011a, 2011b) has also examined how the work of the British meteorologist Gordon Manley, which combined the study of place, people and lived experience of weather, resonated with popular audiences and stands as an example of effective science communication (see also Endfield, Veale, and Hall 2015). Beyond climate, Matless (2017, 372) has proposed the ‘Anthroposcenic’, which holds that landscape, and specifically storytelling about landscape loss, offers a valuable cultural‐historical geographical contribution to Anthropocene discourse. For Matless, scenes such as eroding coastlines and retreating glaciers are emblematic of Anthropocene imaginaries, but also act as a ‘point of access to questions of environmental inheritance, at once local, national, continental, maritime and planetary’ (2017, 372). By elevating storytelling, narrative and art alongside scientific accounts, such efforts to ‘reculture’ the environment have been successful in carving out a space for humanistic scholarship on global problems like climate change. However, there remains much potential to broaden locales and time periods. Equally, geographers could further contribute to understanding of how environmental phenomena were conceptualised by past societies averred to have undergone transformation and/or demonstrated resilience amidst environmental changes and extremes (as argued by Degroot et al. 2021; see e.g., McDonagh et al. 2024), which are being reconstructed with ever greater precision (Section 2).

The expansion of land and seaborne empires precipitated new entanglements between colonial agents, unfamiliar environments, and indigenous populations. There are now excellent macro‐ and micro‐scale studies documenting knowledge production in the imperial and colonial settings out of which disciplines such as botany, biology, medicine and meteorology in no small part emerged (Portuondo 2009; Bleichmar et al. 2008; Cagle 2018; Jones 2022). A key line of historical geographical enquiry has focused on European naturalists in the tropics and the emergence of discourses on tropical climates, health and habitation (Driver and Martins 2005). These were imbricated discourses that not only shaped behaviour but justifications of colonial rule itself (Adamson 2012). Colonial expansion also fostered attention on environmental conditions that were viewed to shape economic activities. In the dry Russian steppe region, soil and its importance for agricultural productivity took centre stage (Moon 2013). This was the context out of which new developments in soil science emerged—knowledge that was later exported to the Great Plains of the United States (Moon 2020). Importantly, Moon's work also situates the environment as a leading character in the historical drama, whereby periods of nineteenth century drought spurred new discourses around climate change and in turn shaped developments in climatology. Elsewhere, histories of meteorology have illustrated how environmental observation and data collection was central to settler colonialism in the US (Grossman 2023), as well as how colonialism shaped the infrastructures of modern climate science (Mahony 2016). Mercer and Simpson (2023) point towards various ways forward in research into imperial and colonial histories of climate and hazards, including how the coloniality of knowledge shapes conceptualisation of such phenomena today.

Supplementing research on the entanglement of science and empire has been a recent rise in work on the erasure of indigenous and local knowledge on the one hand, and indigenous agency and resistance on the other. An array of studies have traced (successful) attempts by colonial scientists, administrators as well as missionaries to devalue and denigrate indigenous and local knowledge, which often had varying logics even if geared towards the overall goal of colonial control (Chambers and Gillespie 2000; Sivasundaram 2005; Carswell 2006; Beattie and Morgan 2021). Elsewhere, research has shed light on the ways through which indigenous and local knowledges shaped colonial scientific activities. Local guides, for example, were central to the interpretation of environmental geographies and evidence of past climatic change (Lehmann 2022; Simpson 2022), while the labour and knowledge of indigenous peoples in settings such as observatories propelled climate data production (Williamson 2021). The ways in which different knowledge systems influence(d) one another should be an important part of future work in the discipline. This promises to be insightful in revealing how ‘different ways of knowing, thinking about, and experiencing the world can and should contribute to policy formulations’ (Offen 2012, 480). The variable traces and representations of indigenous actors in the written record presents challenges; however, widely practised techniques of reading along and against the archival grain offer potential to open up new perspectives, especially outside of the Anglophone world.

Tracing genealogies of environmental scientific knowledge has been another important facet of research. The deeper roots of contemporary climate science and discoveries relating to the Greenhouse Effect have been the subject of books by historians of science (Fleming 1998; Weart 2003). These works tend to centre European and North American (male) scientists such as Svante Arrhenius, John Tyndall, and Guy Calendar at the heart of what Fressoz and Locher (2020, 221, 222) term the ‘official genealogy’ of climate science—a representation that is still prominent in IPCC illustrations of the history of climate science (Mercer and Simpson 2023). Studies at the national level have shown how distinctive representations of the earth's physical systems emerged under the influence of various experiential and contextual factors, including periods of social upheaval (Oldfield and Shaw 2015; Oldfield 2021). The role of contestation and counternarratives in shaping environmental knowledge production is a key strength of geographical research in this domain and has broader potential to provide valuable context for the present. As above, the challenge of unbalanced archival traces creates difficulties in revealing histories of environmental knowledge making beyond the dominant actors in institutional archives. Here, geographers can take inspiration from the series of methodological innovations practised by Mercer (2021), who applied techniques such as robust speculation, upstreaming, and cross‐contextualisation to reveal how gender relations influenced the production and use of atmospheric knowledge in colonial Tasmania (see also Endfield and Nash 2005).

A related set of contributions have advanced genealogies of knowledge production and international policymaking relating to climate change. Drawing from environmental social science and critical physical geography, this work has helped bring to light the framings and priorities underpinning widely used concepts such as adaptation (Schipper 2006; Kythreotis et al. 2024), modes of climatic variability such as El Niño (Adamson 2022, 2023) and the implicit assumptions in policymaking such as the 2°C target (Randalls 2010) and loss and damage (Roberts and Huq 2015). The relevance of this work for policy and practice is relatively easily demonstrated by its direct engagement with widely used and debated concepts and constructs.

## 4 Environmental Impacts and Interventions

A third set of research areas in environmental historical geography fall under the umbrella of material environmental impacts on humans and human impacts on the environment. While often still drawing on qualitative approaches, research in the former realm usually moves away from environmental knowledges to trace tangible events at the environment‐society interface. However, a good deal of work has also explored the multi‐directional relationships between knowledges and materialities (e.g., Endfield and Nash 2002). At the macro level, a largely separate branch of quantitative research using historical big data to test relationships between past climate variability and socio‐economic indicators over the very long run. On the flip side, a longtime focus of historical and environmental geography has been the ways in which society has intervened in and transformed environments. In this domain, there is a large body of research on the legacies of control and intervention in landscapes, which features a strong contingent of perspectives from (post‐)colonial contexts.

Geographical research on environmental impacts has had to grapple with disciplinary legacies of environmental determinism. This not only concerns determinist theories of the late‐19th and early‐twentieth century geographers, but also the ostensibly neo‐determinist and reductionist nature of some work on climate impacts (Hulme 2011). For their part, historical geographers have largely avoided the reductionist trap, not least by centring concepts such as vulnerability, resilience and adaptation at the heart of the analysis, which not only allow for but underscore human agency (Endfield 2007, 2011a, 2011b; Widgren 2012; Hannaford 2014; Adamson 2014). A plethora of research has examined the impacts of environmental extremes and longer‐term changes on historical geographies and socio‐political processes (Mulcahy 2006; Gergis, Garden, and Fenby 2010; Carey 2010; Johnson 2011; Adamson 2014; Rohland 2018; Hannaford 2018; Pribyl et al. 2019; Gooding 2022). The substantive incorporation of theory is a strength of geographical research here, as is the readiness to integrate scientific analyses and environmental reconstruction into the evidence base. This has enabled scholars to link global and local environmental processes as well as bridge the gap between event and structure. All of these studies have shown that the environment mattered in history and that humans shaped the diverse ways in which outcomes unfolded. Degroot et al. (2021), however, point towards an ongoing bias in attention towards cases of collapse rather than of resilience. There is also the potential for more systematic comparative research focusing on the mechanisms via which societies that experienced relatively similar environmental conditions and extremes followed different trajectories and outcomes (van Bavel and Curtis 2016).

Quantitative research on historical environment‐society interactions has attempted to establish causal relationships between climatic changes and large‐scale human activity through correlations and other statistical methods, for example wavelet analysis (Zhang et al. 2007, 2011; Burke et al. 2009; Pei et al. 2014). This work, typically published in high impact scientific journals, has found relatively consistent large‐scale associations between climatic time series and trends in agricultural production, conflict and warfare, epidemics, population, and macro‐economic activity. Aside from critiques of reductionism that emerge from imbuing quantitative (palaeo)climate data with predictive power over human behaviour, these studies tend to be beset by issues stemming from insufficient critical engagement with the content and use of historical (big) data (van Bavel et al. 2019; Degroot et al. 2021). Such methodological issues have been highlighted in studies on the recent past as well, perhaps most notably in terms of sampling bias and the streetlight effect (Adams et al. 2018). Revisiting datasets and revealing the uncertainties and biases within is an important task, as is driving forward efforts in the construction of new multidisciplinary historical datasets, which could include the adoption of citizen science approaches covered in Section 2.

Amongst the richest bodies of research in the field are studies relating to interventions and adjustments to landscape, which have been prominent since the disavowal of environmental determinism. The evolution and effects of policy interventions relating to water is an area that has received particular attention, including responses to wetland drainage, pollution, and place‐based conflict over water resources (Powell 2002; Imlay and Carter 2012; Colten 2012, 2018; Musemwa 2014). Field evidence has also played a key role in investigations of how agrarian change reshaped physical landscapes (Doolittle 2000; Denevan 2001; Whitmore and Turner 1992) and the emergence of ‘landesque capital’ (Håkansson and Widgren 2016). In addition, wider work on global agricultural history has questioned the assumptions in pre‐existing land cover models (Widgren 2018), showing how historical geographical perspectives can inform and refine modelling approaches (Kabora et al. 2024). At the global scale, investigation into the drivers and consequences of plant transfers has provided insight into the timing of botanical (and animal) exchanges (Alpern 2008), as well as the ‘hidden’ agencies of Africans in the transformation of agricultural systems (Carney 2001; Hannaford 2023).

As per research on environmental knowledges, legacies of colonial bordering and intervention have been a central concern, also in historical political ecology (Davies 2007). The long‐term consequences of exclusionary land policies and restrictions on resource use have been a common denominator in this space. This includes the way in which ecological concerns and conservation were used to curb indigenous farming practices and other livelihoods (Sioh 2004; Davis 2004; Andersson, Östlund, and Törnlund 2005; Brooks 2005; Wolmer 2005; Gupta 2009; Musemwa 2009; Barton and Bennett 2010; Ndumeya 2020), or even human settlement altogether (Sunseri 2005). Several features of this work render it amongst some of the most novel historical geographical scholarship. First, in addition to interventions themselves, it often traces resistance and agency by colonised subjects through reading both against and along the archival grain. Second, it makes explicit links between (Western) scientific theorising and colonial narratives about the environment and its material consequences. And third, these works have drawn connections between colonial and postcolonial environmental geographies (Davis 2004; Walker 2015). These features inspire some of the suggestions for future research in the concluding section that follows.

## 5 Conclusion and Future Directions

This article has explored three of the major currents in environmental historical geography: environmental reconstruction, environmental knowledges and ideas, and material impacts and interventions. While the core business of environmental historical geography has remained relatively constant, research within each area has examined a growing number of themes, with a particular recent growth in historical geographies of climate. Historical geographers have also embraced wider theoretical developments and methodological innovations from geography and beyond, which has enabled research to counter reductionist conceptualisations of environment and society. I conclude here by briefly distilling a small selection of future directions for work on environment‐society relations past and present, to which environmental historical geography is well positioned to contribute. Particular emphasis is placed on the intersections between these three overarching areas of research as well as the relevancy question.

The influence of historical big data and AI were discussed in Sections 2 and 4, as were the epistemological tensions that have acted to separate efforts to reconstruct environmental changes and impacts from the themes discussed in Section 3. As these developments continue apace, however, environmental historical geography has much to contribute to interrogating contexts of source and knowledge production. Research in historical geography has been key in unpacking the role of knowledges and discourses in shaping material developments, which is sometimes a missing link in scholarship connecting environmental changes and extremes to human behaviour. Indeed, Offen (2012) highlights the bridging of the ‘ideational‐material spectrum’ as a strength of historical geography. Further work that reveals discursively produced materialities, but also influences in the other direction, remains a promising area of research. Disentangling these multi‐directional interactions can be especially insightful in postcolonial settings, where the complex and entangled legacies of imperial and colonial projects are intellectual as much as physical and structural. It hardly needs stating that an inherent strength of historical geography here lies in moving beyond the very recent past, but this is vital in developing understandings of coloniality that extend back beyond formal decolonisation and to the origins and intensification of imperial and colonial expansion. Where relevant, such efforts should entail integration of timeseries of recurring environmental extremes that form the backbone of much of the work discussed in Section 2. Broadening geographical foci is an obvious area for future work, however there are significant opportunities to develop comparative studies centred around the themes discussed above, which can help enhance the theoretical foundations of the discipline.

A final thought relates to the question of relevancy. There is now burgeoning research on the role of historical perspectives in informing understanding of the challenges associated with environmental change (e.g., Adamson, Hannaford, and Rohland 2018), conceived more broadly (or vaguely) as learning from the past (Mordechai and Tubi 2024). However, as argued by Mordechai and Tubi (2024), examples of research designs that are conducive to such learning are few. Here, again, environmental historical geography is well positioned to contribute through, for example, application of community‐centred and participatory historical approaches, which have been successful in making global narratives around environmental change more meaningful and in building action and resilience (DeLyser 2014; Geoghegan 2014; McDonagh et al. 2023). Collectively, these areas have the potential to further reinvigorate discussions of environment‐society relations in historical geography and beyond.
